# Job satisfaction and career intentions of registered nurses in primary health care: an integrative review

**DOI:** 10.1186/s12875-018-0819-1

**Published:** 2018-08-07

**Authors:** Elizabeth Halcomb, Elizabeth Smyth, Susan McInnes

**Affiliations:** School of Nursing, University of Wollongong, Northfields Ave, Wollongong, NSW 2522 Australia

**Keywords:** Primary health care, Nursing, Workforce, Job satisfaction, Retention, Career intention

## Abstract

**Background:**

There has been a significant growth of the international primary health care (PHC) nursing workforce in recent decades in response to health system reform. However, there has been limited attention paid to strategic workforce growth and evaluation of workforce issues in this setting. Understanding issues like job satisfaction and career intentions are essential to building capacity and skill mix within the workforce. This review sought to explore the literature around job satisfaction and career intentions of registered nurses working in PHC.

**Methods:**

An integrative review was conducted. Electronic databases including: CINAHL, MEDLINE, Scopus and Web of Science, and reference lists of journal publications were searched for peer-reviewed literature published between 2000 and 2016 related to registered nurse job satisfaction and career intentions. Study quality was appraised, before thematic analysis was undertaken to synthesise the findings.

**Results:**

Twenty papers were included in this review. Levels of job satisfaction reported were variable between studies. A range of factors impacted on job satisfaction. Whilst there was agreement on the impact of some factors, there was a lack of consistency between studies on other factors. Four of the six studies which reported career intentions identified that nearly half of their participants intended to leave their current position.

**Conclusion:**

This review identifies gaps in our understanding of job satisfaction and career intentions in PHC nurses. With the growth of the PHC nursing workforce internationally, there is a need for robust, longitudinal workforce research to ensure that employment in this setting is satisfying and that skilled nurses are retained.

## Background

The recruitment and retention of nurses is problematic worldwide. There is a maldistribution of human resources for health, a shortage in the overall number of qualified nurses and an aging nursing workforce [1]. Job satisfaction has been cited as an important factor contributing to the turnover of nurses and as an antecedent to nursing retention [2–4]. Therefore, understanding factors that impact on job satisfaction is important to inform recruitment and retention strategies.

The concept of job satisfaction is multifaceted and complex. Job satisfaction has been the focus of much research around organisational behaviour. Lu, et al. [5] define job satisfaction as not only how an individual feels about their job but also the nature of the job and the individuals’ expectation of what their job should provide. To this end, job satisfaction is comprised of various components, including; job conditions, communication, the nature of the work, organisational policies and procedures, remuneration and conditions, promotion / advancement opportunities, recognition / appreciation, security and supervision / relationships [5]. Whilst levels of job satisfaction vary, several common factors emerge across studies [6, 7]. These include working conditions and the organisational environment, levels of stress, role conflict and ambiguity, role perceptions and content and organisational and professional commitment [5–8]. Given these factors it becomes clear that research about job satisfaction cannot be undertaken across the nursing profession as a whole, but rather needs to consider various settings and organisational environments to understand the issues facing different nursing groups.

Career intentions can be described as the intention to leave ones’ job voluntarily [9]. This process may start with a psychological response to negative situations in the workplace or undesirable aspects of the job. Subsequently, a cognitive decision is made to leave the position and withdrawal behaviours occur as the person moves out of the workplace [10]. Like job satisfaction, a number of common determinants for career intention have been identified. These include organisational factors, management style, workload and stress, role perceptions, empowerment, remuneration and employment conditions and opportunities for advancement [10]. In several studies, job satisfaction has been shown to impact on career intentions [11, 12].

Despite the common themes in this workforce literature, much of the research around job satisfaction and career intentions reported to date has focussed on acute care nurses [2, 5, 6, 10, 11, 13, 14]. Given the impact of organisational factors, roles and employment conditions it is important to consider different groups of nurses, such as those employed in PHC, who are employed in settings unlike those of their acute care colleagues. PHC nurses practice in a range of settings, including general practices, schools, refugee health services, correctional settings, non-government organisations and community health centres [15]. As such, their employment conditions and work environments are unlike those of acute care nurses who are employed by large health providers or government funded health services (17). The small business nature of primary care in many countries and the predominance of charities and non-government health providers makes employment in the PHC setting unique [16–18]. Lorenz and De Brito Guirardello [19] describe the PHC work environment as “not always favourable to the professional practice of nurses”(p. 927), citing lack of equipment, inappropriate physical environment and occupational risks as key contributors to dissatisfaction. Additionally, there are significant difference between the roles, responsibilities and work environments of acute and PHC nurses [20]. These differences and the impact of such factors on job satisfaction and career intentions mean that acute care nursing workforce research cannot be simply generalised to the PHC setting. With the growth in the PHC nursing workforce and the need for a strong nursing workforce in this setting it is timely to explore the job satisfaction and career intentions of PHC nurses. Therefore, this review sought to critically synthesise the literature around the job satisfaction and career intentions of registered nurses working in PHC.

The underlying research questions are:

- What was known about the main outcomes of studies regarding PHC registered nurses job satisfaction?
- What was known about the career intentions of PHC registered nurses?

Registered nurses are the focus of the review as they are the largest nursing workforce in PHC [21].

## Methods

### Design

This integrative literature review is informed by Whittemore and Knafls [22] framework. It provides a thorough examination of the existing literature following the five stages of review: problem identification, literature search, data evaluation, data analysis and presentation [22].

### Search strategy

A systematic search strategy was designed to guide the search of electronic databases: CINAHL, MEDLINE, Scopus and Web of Science. Key search terms included; nurs*, primary health care, community care and job satisfaction or career intention. The search was confined to English language peer reviewed papers of original research. Given the significant changes in PHC systems internationally, only papers published between January 2000 and 2016 were considered. The reference lists of publications were also reviewed to identify further literature.

### Inclusion criteria

Table 1 details the inclusion and exclusion criteria. Papers were excluded if they focussed on a particular nursing specialty (e.g. community mental health nurses) or were based in residential care settings (e.g. nursing homes), as the issues with this workforce are somewhat different to other PHC settings. Studies that focussed on nurse practitioners and/or advanced practice nurses (e.g. [23]), or specifically on nurse managers were excluded as these nurses may have different perceptions and experiences to registered nurses. Remoteness itself was not considered to constitute PHC nursing, therefore, papers focussed on rural or remote nurses without being specifically PHC focussed were excluded. Research articles were also excluded if the findings did not isolate PHC nurses from acute care nurses or other health professionals.

**Table 1:** Inclusion / Exclusion Criteria

Inclusion Criteria	Exclusion Criteria
• Published between 2000 and 2016. • Written in English language. • Peer-reviewed original research. • Explores issues related to job satisfaction and the retention of registered nurses employed in PHC settings.	• Literature reviews, discussion papers, dissertations and theses. • Papers focussed on advanced practice nurses / Nurse Practitioners. • Papers focussed on nursing speciality areas. • Data about nurses aggregated with other nursing specialties and health professionals. • Nurses employed in residential settings.

### Study selection

**Fig. 1:**
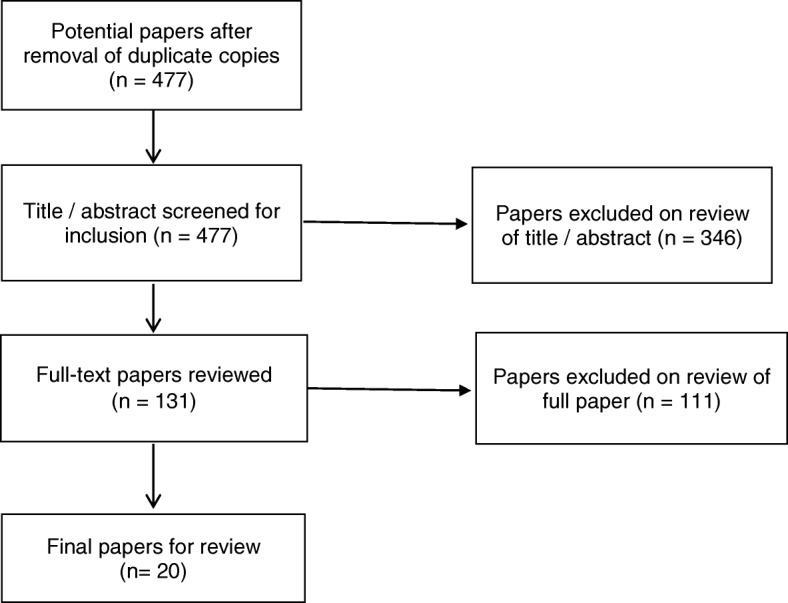
Process of paper selection – Prisma Flow diagram [24]

After removal of duplicates, 477 citations were yielded from the search. These citations were exported to Endnote X8™ for review of their titles, followed by closer evaluation of the abstract. This process identified that 346 papers did not meet the inclusion criteria, leaving 131 papers where the full-text was retrieved. Of these papers, 111 did not meet the inclusion criteria, and so were excluded. This left 20 papers for inclusion in the review. (Fig. 1).

### Appraisal of methodological quality

Determining the methodological quality of the included studies was difficult due to the broad sampling frame and various research designs [22]. As identified by Whittemore and Knafl [22], there is no gold standard for evaluating quality in research reviews. In this review we conducted quality appraisal using the tool provided by the Center for Evidence Based Management [25]. The major areas of concern were around the quality of reporting of the instrument development and validity / reliability measures in some papers [26–31]. Given the relatively small number of included papers and the minor nature of the limitations identified none of the studies were excluded based on their methodological quality.

### Data abstraction and synthesis

Once the included papers were identified all data was abstracted into a summary table. The main characteristics that were extracted included;

- Citation
- Country
- Study design
- Sample
- Study aim
- Methods
- Main outcomes related to job satisfaction or career intention

The nature of the included papers, in terms of the heterogeneity of the measures used, meant that thematic analysis was the most appropriate technique for aggregating the findings. Therefore, data is presented in a narrative form around the key themes that emerged from the literature.

## Results

Of the 20 included papers (Table 2), 15 (75%) described quantitative studies, 4 (20%) papers described qualitative projects, and the remaining paper (5%) employed a mixed-method approach. Most of the included papers reported research undertaken in Canada (*n* = 8, 40%), with other studies coming out of the United Kingdom (*n* = 4, 20%), the United States of America (*n* = 5, 25%), one paper each from Saudi Arabia, South Africa, and Brazil.

**Table 2:** Summary table

Reference	Aim	Country	Sample	Methods	Findings
Almalki et al. [42]	Examine the relationship between quality of work life (QWL) and turnover intention	Saudi Arabia	508 PHC nurses (87% response)	Survey Brooks Quality of Nursing Work Life Anticipated Turnover Scale	• 67.3% female. 44% aged between 20 and 29 years. • Mean time in current PHC organisation 6.6 years and mean 6.1 years in current position. • Brooks’ scale can range from 42 to 252. Participants scored from 45 to 218 (mean 139.45), indicating they were dissatisfied with work life. • 40% respondents indicated a desire to leave their current PHC workplace. • Turnover was significantly related to quality of work life, explaining 26% of variance (*p* < 0.001). • Quality of work life and demographics explained 32.1% of variance (*p* < 0.001). • In the final model, work context (*p <* 0.001), duration in positional (*p* < 0.05), payment per month (*p <* 0.05), and gender (*p <* 0.05) were statistically significant.
Armstrong-Stassen [26]	Compare work-related concerns, job satisfaction, and factors influencing retention	Canada	1044 PHC nurses (52% response)	Survey	• 98% female. Mean age 44 years • Employed in the position and agency for a mean of 8 years. • 4/5 highest ranked concerns were the same across the 3 settings, namely; inadequate staffing, increasingly complex needs of clients, working with vulnerable families with many problems, and dealing with difficult clients. • There were significant differences for 15 of the 17 work-related concerns between nurses from the 3 settings (*p* < 0.001). • Community Care Access Centre nurses expressed greatest concern about the emotional effects of the job. HCNs reported significantly greater concern over working conditions and safety issues. PHNs reported significantly greater concern about poor facilities. • There was a significant difference between the three groups for 6 of the 7 job satisfaction items (*p <* 0.001). • 3 /5 highest ranked retention items were identical across the three settings, although the ranking order varied.
Best and Thurston [33]	Test standardised job satisfaction tool	Canada	PHC Nurses Pre: *n* = 44 (60% response). Post: *n* = 43 (49% response)	Survey Index of Worklife Satisfaction (IWS)	• Most important components of job satisfaction were autonomy then pay. • Participants most satisfied with professional status. • Satisfaction with professional status and interaction significantly increased over the study (*p* < 0.01). • Aspects of work life giving the most satisfaction were quality client care / making a difference. Other factors identified included; autonomy/independence, colleague relationships, and opportunities for health teaching, respect/recognition, and client appreciation. • An open-ended question asking about what respondents would change identified administrative concerns, the need for more educational opportunities, more time for client care, more respect/recognition, and more opportunity for independence/autonomy.
Betkus and MacLeod [43]	Examine job and community satisfaction; how these relate to retention	Canada	124 PHC nurses in rural and small urban communities (76% response)	Survey Piedmonte’s Work Satisfaction Index (WSI)	• 99% female. Mean age 43 years, 67% aged over 40 years. • 60% were first licensed ≥20 years ago, 50% had worked 5 years or longer in PHC nursing, and 49% were in PPT positions • Overall, most were satisfied. Most satisfied with professional status, professional interactions, and autonomy. Least satisfied with salary. • PHC nurses had varied perceptions of the organisational environment. • Nil correlation between age and job satisfaction (*p* > 0.05). • 52% indicated intent to stay in position for 5 years or more; • No correlation between job satisfaction and retention. • 52% intended to stay in their job for ≥5 years. 28% planned to leave within 2 years – this was 43% of those aged ≤35 yrs. • Factors impacting on decisions to stay were age, retirement, family circumstances and the economy. • Job satisfaction and community satisfaction were correlated (*p <* 0.001).
Campbell, et al. [34]	Impact of organisational structure on job satisfaction	USA	192 PHC nurses (55% response)	Survey Alexander Structure Instrument (ASI) McCloskey / Mueller Satisfaction Survey (MMSS)	• 96.9% female. 40.5% aged between 41 and 50 years. • 85.9% worked full-time, 48% had worked in nursing ≥20 years, and 40% had been employed in their department < 5 years. • The more that supervisors and subordinates work together concerning tasks and decisions and the more that individuals are involved in decision making and task definition, the higher job satisfaction. • Increased vertical participation and horizontal decision-making opportunities equate to higher job satisfaction. • Full-time staff reported higher levels of vertical participation compared to part-time staff (*p* < 0.002). • ‘Formalization’ (i.e. standardised policy, practices and position responsibilities) was not significantly related to job satisfaction. • Significant differences found between position classifications for total ASI score (*p* = 0.000), and vertical and horizontal participation subscales (p = 0.000). • Global job satisfaction scores ranged from 75 to 144 (Mean 113.04; SD = 6.32). As 94 is the lowest score that indicates satisfaction, most participants were satisfied. • No significant differences for job satisfaction were associated with current position or primary work assignment. • Educational preparation made a difference to job satisfaction with MSN prepared nurses (8.5%) scoring highest on MMSS. • To make the job more satisfying 27% indicated “better pay”, 19% wanted increased management feedback and staff recognition, 15% indicated a desire to have more input and decision-making opportunity in their jobs, and 4% sought to increase role clarity. • 98% of participants planned to remain working in their health department. • Enjoyment of what they do, autonomy, flexibility, scheduling, benefits, and low stress were reported as intention to remain working.
Cameron, et al. [35]	Nurse satisfaction and retention in hospital and community settings	Canada	644 Community nurses (54% response)	Survey	• Most participants female (97.5%), Mean age of 45.07 years and 60% work full-time • Community nurses were significantly more likely than hospital nurses to report greater cohesiveness in the work place and a higher degree of support from supervisors related to feedback and recognition. • Community nurses were significantly more likely than hospital nurses to report higher autonomy and greater satisfaction with work demands. • Hospital nurses were significantly more likely than community nurses to be satisfied with remuneration.
Curtis & Glacken [37]	Level of and factors affecting job satisfaction	Ireland	351 PHC nurses (35.1% response)	Survey (IWS)	• 35% aged 36–45 years. 34.5% practiced as PHN 1-to-5 years • 53.3% hadn’t worked as part of a primary care team previously. • IWS score of 12.62 (range 0.5–39.7) suggests a low level of job satisfaction. • Variables considered most important to their job satisfaction were: autonomy, interaction and pay. Task requirements were rated as least important to their job satisfaction. • Statistically significant differences in IWS scores noted for 3 age groups (*p* < 0.05): < 35 years, 35–45 years, and 45 years (p = 0.000). • The > 45 years age group were significantly more satisfied than younger colleagues. • There was no significant difference in job satisfaction between the < 35 years group and the 35–45 years group (*p* = 0.574). • Those employed as PHNs for > 10 years had a significantly higher job satisfaction compared to those with < 5 years (*p* = 0.001) and 6–10 years experience (*p* = 0.006). There was no significant difference between participants with < 5 years and 6–10 years experience (*p* = 0.995). • There was no statistically significant difference between job satisfaction and educational background (*p* = 0.478), rurality of practice (*p* = 0.137) or if participants were members of a constituted primary care team (*p* = 0.16).
Cole, et al. [36]	Difference in job satisfaction between nurse managers and nurses	Canada	88 PHC nurses (20 managers and 68 staff nurses) (56% response)	Survey Stember’s model of job satisfaction	• 94% female, 75% worked full-time and 75% had flexibility in work schedules. • Job experience: Managers’ 20–42 yrs. (mean 29.7 yrs); Staff nurses 1–50 yrs. (mean 21.6 yrs). • Both managers and staff reported high job satisfaction – a mean > 3 for each subscale. • There was no significant differences between managers and staff nurses on the total job satisfaction scores (*p* = 0.530). • Managers were significantly less satisfied than staff nurses in both the ‘influence’(participation in decision making) (*p* = 0.026) and ‘interpersonal relationship’ (*p* = 0.008) subscales. • The comparison of education levels and job satisfaction was inconclusive.
Delobelle et al. [2]	Examine the relationship between job satisfaction, turnover intent and demographic variables	South Africa	143 Rural PHC nurses (82% response)	Mixed-methods Survey & focus group	• 87% female, 58% were aged > 40 yrs. • 83% had been working in the unit for 10 years or less. • 51% of participants considered turnover within 2 years. • Job satisfaction was reportedly moderate (Mean = 3.2; SD 0.5). • Higher mean scores were attained for work itself and co-workers, and lower scores for pay and work conditions. • There was a significant difference in job satisfaction amongst professional ranks - NA and ENs were more satisfied than RNs (*p* < 0.001) • Job satisfaction negatively correlated with unit tenure (*p* < 0.05), professional rank (*p* < 0.01) and turnover intent (*p <* 0.01). • There was no significant difference between job satisfaction and age, education or years of nursing. • Turnover intent was statistically significantly explained by job satisfaction, age and education (*p <* 0.001). • Younger and more highly educated nurses are more likely to show turnover intent. • Nurses who reported more satisfaction with supervision were nearly 40% less likely to consider a job change. • The most satisfying aspects of job were the nature of work itself, staff and patient relationships, adequate staffing and resources. • The least satisfying aspects of the job were; work conditions (including lack of space), adequate staffing, lack of equipment and supplies, inadequate security, high workload, and the time spent doing non-nursing activities. Participants were also dissatisfied with their pay and benefits, lack of training and promotion, and lack of recognition and support from supervisors. • When asked what factors would help in their work respondents identified work conditions and equipment (88%), improved pay (69%), additional training (60%) and more staffing (49%).
Doran et al. [38]	Relationship between employment, job satisfaction and perceived job security	Canada	700 HCNs (479 RNs, 211 RPNs, and 9 APNs) (49.0% response)	Survey Nursing Job Satisfaction Scale	• 98% female, mean age 45 years, mean 8.2 years of community experience, 30% full-time. • A mean score of 3.84 (SD = 0.54) demonstrated that participants were moderately satisfied with work enjoyment. • Of the items measuring work enjoyment, participants were least satisfied with the conditions of the job (1.69) and balance between work and leisure (2.14). A mean score of 2.62 (SD = 1.28) indicated a low level of satisfaction with job security. • There were significant differences in nurses’ work enjoyment between agencies (*p <* 0.05). • Older nurses rated work enjoyment higher than younger nurses. • There were significant differences in participants’ satisfaction with time for care among agencies (*p <* 0.05). • Participants who had been employed by the same agency for a longer period were less satisfied with time for care than those who had been employed by the same agency for a shorter period. • Participants paid on an hourly basis were more satisfied with their time for care than those paid on a per-visit basis. • Participants who were employed on a casual basis perceived less job security than those employed full-time.
Flynn and Deatrick [27]	Identify attributes important to professional practice and job satisfaction	USA	58 HCNs	Focus groups	• 91% female, Mean age 44.7 years, and had worked in HC 7 years or longer (50%). • Continuing education opportunities were identified by all to enhance retention. • There were 6 major categories and 8 sub-categories identified to positively influence and support job satisfaction and retention if present or working towards; – ‘An extensive, preceptor-based orientation’ – ‘An organised and supportive office environment’ including; Real-time phone support, Interdisciplinary coordination and follow-up and Adequate and efficient clerical assistance – ‘Reasonable working conditions’ including Realistic workload, Adequate staffing and Scheduled days off – ‘Accessible field security’ – ‘Competent and supportive management’, including; Competent nursing supervisors and Supportive administrative practices – ‘Patient-centred mission and values’
Graham et al. [39]	Examine relationship between autonomy, control-over-practice, workload and job satisfaction	Canada	271 PHC nurses (79.7% response)	Survey	• Mean age 42.5 years, 52% permanent full-time, 50% worked in PHC for < 7 years. • 53.5% reported being very satisfied with their jobs. • Control-over practice (*p* = 0.01) and workload (*p* < 0.01) were reliable predictors for job satisfaction. • As workload increased job satisfaction decreased. • Increases in control over practice scores were related to increased job satisfaction. • Interaction between autonomy and workload was a significant predictor for job satisfaction (*p* < 0.01). • The interaction between age and workload was a significant predictor for job satisfaction (*p <* 0.01).
Junious, et al. [28]	Explore job satisfaction and changes needed to boost levels of job satisfaction	USA	71 School Nurses (78.9% response)	Focus Groups	• All female, 55% worked in an elementary school, 84% had ≥3 years’ experience. • 83% of participants reported being satisfied with their job. • 17% were dissatisfied with their job, primarily related to poor remuneration and lack of trust / support from administration. • Four major themes arose: (a) benefits, (b) resources, (c) autonomy, and (d) coping • Theme 1 Benefits. Issues related to job satisfaction included things such as creativity, freedom, growth, power, work standards, and ethics. Participants were very satisfied with job flexibility and paid holidays. • Theme 2 Resources. Resources, such as salary and supplies, were areas where participants were least satisfied with their jobs. Participants also wanted to be appreciated, valued, and compensated fairly for job performance. • Theme 3 Autonomy. Autonomy was considered the ability to act independently within one’s scope of professional practice. When autonomy was not expressed, “isolation” emerged as the divergent theme. Over half of the participants stated that working with outside agencies increased satisfaction (53%). • Strategies that could be implemented to increase satisfaction included; career or pay scale differentiating qualifications (52%), increased professional development (32%), supervision by another nurse rather than nonnurse (24%), and a designated budget / supplies (17%). • Factors that negatively impacted on job satisfaction were; uncooperative staff and parents (61%), constant interruptions (48%), and the expectation that they would use their personal vehicle for work (13%).
Lorenz & Guiradello [19]	Relationship between burnout, satisfaction at work, quality of work and the intention to quit	Brazil	168 PHC nurses (58.5% response)	Survey Nursing Work Index-Revised Maslach Burnout Inventory	• 88.4% female, Mean age 36.3 years, 6.6 years’ experience in primary care; employed 4.9 years at current job. • Satisfaction measure on Likert scale. 62.6% considered that they were satisfied at work. 34.9% were dissatisfied. • Satisfaction at work was significantly related to organisational support (*p <* 0.01) and control over the practice environment (*p <* 0.01). • The intention to quit their job was significantly related to autonomy (*p <* 0.01)
O’Donnell, et al. [41]	Degree of professional support felt by PHC nurses and their career intentions	Scotland	200 PHC nurses (61% response)	Survey	• All female, 49% aged 40–49 years and 29% were aged > 50 years. Employed as PHC nurse for a mean of 10 years (Range 0.5–24.0 years). • 15.5% intended to leave general practice in the next 5 years. • There was a significant association between age and intention to leave employment (*p* = 0.001), with 60% of those intending to leave aged ≥50 years, although 40% were aged under 50 years. • Isolated nurses are less likely to intend staying in practice nursing (*p* = 0.009). • 52.3% felt isolated at least sometimes, 43.7% reported feeling isolated sometimes, and 31% of nurses worked alone. • 77.3% of isolated nurses intended to continue working for the coming 5 years compared to 91.4% of non-isolated nurses. • Factors contributing to feelings isolation are generally located in the work environment. • Training and qualifications being used to the full and a productive appraisal both significantly reduce feelings of isolation, as did the intention to continue working in the future.
Royer [32]	Perceptions of work and workplace to identify factors affecting tenure intent	USA	478 C/PHC nurses (76% response)	Survey TCM Work Commitment Survey	• 73% clinical nurses, 11.6% were in management/administrative positions and 22% supervisors • 70.5% of respondents were middle aged or nearing or at retirement age • 1/3 were either thinking about leaving, looking into leaving, or planning to leave the job in 1 year • Of the 70% of respondents aged > 45 years, 1/3 were planning to leave within 1 year. • 46% of those aged 35–45 years were looking into leaving, and almost 40% of those aged 56–65 were thinking about leaving. • Respondents aged 35–45 years are 4.3 times more likely to be looking into leaving compared with those nurses who are older. • Respondents who have the least tenure (1–36 months) are 0.35 times less likely to be planning to leave < 1 year than those with greater tenure. • Respondents who have increasing attachment (affective commitment) to the job are also 1.7 times more likely to be looking into leaving and three times more likely to be planning to leave within 1 year than those who are committed in other ways. • Respondents who hold obligatory or loyalty commitment (normative) to the job are 1.4 times more likely to be planning to leave within 1 year than those who are committed by attachment or cost.
Storey et al. [29]	Impact of age on retention	England	485 PHC nurses (61% response)	Survey	• 78% respondents were aged between 40 and 59 years. 47% worked full-time. • 178 district nurses, 114 health visitors, 56 school nurses, and 137 practice nurses • 61% indicated that their role lived up to expectations. There was no significant difference across professional groups. • Older nurses are more likely to report that their role lived up to expectations opposed to younger ones (p = 0.001). • There was no difference in happiness in their current role between those aged under and over 50 years. • Older nurses were more likely to report being happy working in nursing than those < 50 years (*p* = 0.006). • Stress and job satisfaction were identified as key factors contributing to respondents views of working as a nurse. • School nurses were significantly less happy than other groups in their current role (*p* = 0.006). • Sources of unhappiness were identified as excessive workload, low morale, disillusionment, high administrative workload, perceived lack of support and staff shortages. • In terms of job satisfaction, ‘relationships with other people at work’ (62%), ‘the actual job itself’ (60%),’ the level of job security in your present job’ (55%) were highest scored. • There was a statistically significant difference between those aged under and over 50 years on nine items related to job satisfaction. • There was a statistically significant difference between those aged under and over 50 years on nine items related to factors encouraging them to stay. • Highest scored scales of dissatisfaction related to salary relative to experience (27%), change management’ (21%), and organisational communications (18%). • Enhanced pay is a factor encouraging retention (*p* = 0.044) for those with degree-level qualifications. • Significant potential causes of nurses leaving were high administrative workloads, problems in combining work and family commitments (*p* > 0.001), and lack of workplace support (*p* = 0.029).
Stuart et al. [30]	Workload management, job satisfaction and challenges	Scotland	31 district nurses	Focus groups & interviews	• Most job satisfaction is derived from the ‘hands-on nature’ of patient care using clinical knowledge and skills. • Nurses liked the ‘personal nature’ of caring for patients in this setting and the formation of ongoing and sometimes intergenerational relationships. • Job dissatisfaction arises with overwhelming workloads, increased time pressure and policy change that negatively affects patient care and feeling devalued. • Nurses are dissatisfied as administrative tasks are taking them away from patient care.
Tourangeau [31]	Factors affecting intention to remain employed	Canada	50 PHC nurses	Focus groups	• 6 categories were found to influence nurse intention to remain employed: I. Job characteristics: variation in clientele and wide use of nursing skills, autonomous nature of work, decision authority; II. Work structures: continuity of care, appropriateness of client expectations, and flexibility in scheduling work hours, workload and use of technology. III. Relationships and communication: clients and families, physicians nursing colleagues, supervisors, CCAC case managers; IV. Work environment: professional practice environment: orientation, education and training; physical work environment: travel demands, access to resources and personal safety; V. Nurse responses to work: work-life balance, meaningfulness of work. VI. Employment conditions: employment status, union status, pay and benefits, unpaid work, work-related expenses, and income stability; • Job satisfaction was not a reported concept affecting intention to remain employed.
Tullai-McGuinness [40]	Predictors of job satisfaction	USA	201 PHC nurses (42.5% response)	Survey Nurse Work Index-Revised Global Appraisal of Autonomous Practice	• Mean age 45 years. Mean experience 17.8 years, with a mean of 8.3 years HC experience. 75% employed fulltime. • Almost 77% of HC nurses were satisfied (ratings > 60). • Diploma nurses had lower satisfaction (69.25%), compared to baccalaureate (74.43%) and associate degree (75.21%) nurses (*p* > 0.05). • There was an inverse relationship between years worked as a home healthcare nurse and satisfaction (*p* < 0.01). • Controlling for years of experience significant predictors of satisfaction were control over practice decisions and practice setting decisions.

The sample sizes of included studies varied from 31 [30] to 1044 participants [26]. Participants spanned the scope of PHC and included community nurses, primary health nurses, general practice nurses, school nurses, and district nurses. In some studies the data from various primary care nursing groups was reported in an aggregated form [32], whilst in other papers there was an attempt to tease out the differences between groups [26, 29].

Eleven (55%) papers focussed on job satisfaction only [27, 28, 30, 33–40], and three (15%) papers reported only data on career intention or turnover [31, 32, 41]. A further six (30%) papers combined measures of job satisfaction and career intention within the same study [2, 19, 26, 29, 42, 43].

The key features and predominant findings of papers are summarised in Table 2. Five overarching themes emerged, namely; levels of job satisfaction, factors that enhanced job satisfaction, factors that reduced levels of job satisfaction, career intentions, and, factors that impacted on career intentions.

### Levels of job satisfaction

The variation in measurement of job satisfaction across studies and the differences in respondent characteristics makes comparison difficult. Most tools measured job satisfaction quantitatively using a Likert scale (agree to disagree) [2, 19, 26, 37–39], whilst one study used qualitative data collected from focus groups and interviews [28]. Studies measured different aspects of job satisfaction including; overall satisfaction (enjoyment, pride), specific aspects of the job (pay, rewards, resources, task requirements, work conditions, training, quality of care, time) and supervision (authority, autonomy, feedback, appreciation, organisational policies, interaction).

In some studies just over half of the respondents were reported to be satisfied with their job [19, 39], whilst in other studies a greater majority indicated that they were satisfied [28]. A small number of studies reported moderate [2, 38] to low levels of satisfaction [37, 42]. Those studies which reported lower levels of satisfaction used more items to measure satisfaction (42 items and 80 items respectively) [37, 42], compared to studies reporting high levels of satisfaction which used only 4 items [19, 39].

### Factors influencing job satisfaction

The ten studies which explored the relationship between job satisfaction and demographics / professional variables demonstrated significant variation [2, 19, 29, 34, 36–40, 43]. Whilst two studies found that age had no significant impact on job satisfaction [2, 43], three others demonstrated that older nurses were more satisfied than their younger colleagues [29, 37, 38]. Similarly, there were variable findings related to the impact of education, with three papers finding no relationship with job satisfaction or inconclusive findings [2, 36, 37], and two papers demonstrating that nurses with higher educational qualifications had reported higher work satisfaction [34, 40]. In contrast, Delobelle et al. [2] found that Nursing Assistants and Enrolled Nurses were more satisfied than Registered Nurses.

Curtis and Glacken [37] reported that those employed for over 10 years had a significantly higher level of job satisfaction than other nurses. However, other studies reported an inverse relationship between years worked in PHC and satisfaction [40] and no significant differences between satisfaction and years of nursing [2].

Other factors that positively contributed to satisfaction included control over clinical practice and decision-making [19, 34, 39, 40], community satisfaction [43], organisational support [19], remuneration [38], and workload [39].

There was significant agreement between studies in terms of the factors that contributed positively to job satisfaction. These included the professional role, respect and recognition from clients and managers, workplace relationships, autonomy, access to resources and the flexibility of the role [2, 27–31, 33, 34, 37, 43].

### Factors negatively impacting job satisfaction

There was a high level of agreement amongst included studies about factors that negatively impacted respondents’ levels of satisfaction. Seven studies identified concerns about adequate remuneration [2, 28, 29, 34, 35, 37, 43]. When comparing hospital and community nurses, Campbell, et al. [34] identified that hospital nurses were significantly more likely than community nurses to be satisfied with their pay.

Another key factor identified in several studies related to the time pressures and high administrative workloads that impact on patient care [2, 26, 30, 33, 37]. Other factors identified to negatively impact job satisfaction included; a lack of recognition [2, 28, 33, 34], poor role clarity [30, 34, 37] and poor organisational communication [29, 34].

### Career intentions

The included studies present an important picture around career intentions. However, caution needs to be applied in the interpretation of these data, as most studies comprise of an ageing workforce who will naturally retire in the near future. Six studies sought to explore the factors impacting on retention [2, 32, 34, 41–43] The highest reported career intentions was reported by Delobelle, et al. [2] with half of all nurse participants (*n* = 69; 51.1%) considering leaving PHC in the next 2 years. Both Betkus and MacLeod [43] and Almalki, et al. [42] also reported that nearly half (48 and 40%) intended to leave their current PHC job in the next year. Royer [32] similarly identified that some 46% of participants aged 35–45 years were considering leaving, and almost 40% of those aged 56–65 were thinking about leaving. The remaining two studies reported that few participants intended to leave their current position [34, 41].

The findings of the three studies which explored job satisfaction and quality of worklife [2, 42, 43], lacked consistency. Almalki, et al. [42] demonstrated that quality of worklife was significantly related to turnover intent (*p* < 0.001), however, this only explained 26% of the variance and was not included in the final model. Whilst Betkus and MacLeod [43] reported no correlation between job satisfaction and retention, Delobelle, et al. [2] found that turnover intent was significantly explained by job satisfaction, age and education (*p* < 0.001). Other factors that were identified as having an impact on career intentions included gender [42], work environment [42], remuneration [42], education [2, 41, 42], satisfaction with supervision [2], feelings of isolation [41], length of time in position / years of experience [32, 42].

## Discussion

This review provides the first synthesis of the literature around job satisfaction and career intentions of registered nurses working in PHC. Given the differences in organisational context, employment conditions and practice environment that likely impact job satisfaction and career intention [17–19] it is important that this group are explored beyond the context of the broader nursing workforce. Considering the imperatives to grow the workforce in PHC settings, to meet community demand, understanding this literature is important to inform both practice and policy. Dissatisfaction with nursing employment is reported in the broader nursing workforce literature. In their survey of 33,659 medical–surgical nurses across 12 European countries, Aiken, et al. [44] concluded that more than one in five nurses were dissatisfied with their employment. The variation in job satisfaction identified in this review highlights the need for further large well-designed longitudinal investigations of the PHC nurse workforce to monitor workforce issues, such as satisfaction and career intentions, over time. Given the links between nurse satisfaction and both retention and patient outcomes [44], this issue should be prioritised.

Our review demonstrated agreement between studies in terms of the positive impact of a professional role, respect, recognition, workplace relationships and autonomy upon job satisfaction. This is consistent with the acute care nursing literature where modifiable factors within the workplace have been demonstrated to influence both job and career satisfaction [45]. In their study, Nantsupawat, et al. [46] demonstrated that job dissatisfaction and intention to leave were significantly lower in nurses who worked in a better work environment. Similarly, in their systematic review, Cicolini, et al. [14] found a significant link between nurses empowerment and satisfaction. The significant role of such modifiable factors highlights an opportunity for managers, employers and policy makers to implement strategies which can improve the workplace and, subsequently, enhance satisfaction.

A key finding of this review was the negative impact of poor remuneration on job satisfaction. Whilst concerns about pay have been previously identified in the acute sector [44, 47], the challenge of lower rates of pay in PHC compared to the acute sector has long been reported [17, 48]. This review adds to the evidence-base around the impact of this disparity on the PHC nursing workforce and highlights the significant implications of not addressing this issue.

Our review also revealed that in many studies large numbers of nurses were intending to leave PHC employment in the near future [2, 42, 43]. This clearly has significant implications for the workforce and service delivery. However, measures of the factors affecting career intentions were variable across included studies as were findings. The difficulties in synthesising such disparate data have been previously identified in the acute care literature [13]. Despite this, there were clear similarities between our review and the broader literature around nurse turnover and intention to leave. In their systematic review of nurses intention to leave their employment, Chan, et al. [13] identified that intention to leave was impacted by a complex combination of organisational and individual factors. Organisational factors included the work environment, culture, commitment, work demands and social support. In contrast, individual factors related to job satisfaction, burnout and demographic factors. The complex interplay of multiple factors that underlie retention is probably the reason that retention is the highest when interventions such as mentoring and in-depth orientations are used to support staff [49].

In their study of acute care nurses Galletta, et al. [50] conclude that the quality of relationships among staff is an important factor in nurses’ decisions to leave. Interprofessional relationships in PHC have long been identified as presenting unique challenges [48, 51]. The complex environment of PHC, whereby services are funded by small businesses or non-government agencies [52], combines with the relatively rapid shift towards interdisciplinary care to create challenges for staff in developing positive relationships [53]. The importance of positive relationships, respect of roles and recognition of value between co-workers demonstrated in our review highlights the value of further work to enhance interprofessional collaboration.

## Limitations

Whilst this review synthesised the available literature, the variation in measurement instruments and sample sizes made comparison difficult. Since not all papers reported the reliability or validity of the instruments they used it is possible that these instruments had issues in their validity. The data presented, however, represents the best available evidence to address the research question.

A further limitation is the variation between PHC settings and international PHC systems that makes comparison difficult. Whilst this review has included all papers written about PHC nurses internationally, local variations mean that care needs to be taken when generalising findings to other contexts, even within PHC.

## Conclusion

This review has identified some key factors that impact on both job satisfaction and career intentions amongst PHC nurses. The importance of the work environment and workplace relationships highlights the need to implement strategies that enhance modifiable workplace factors. The numbers of nurses across studies indicating an intention to leave is a significant concern at a time when we need to build the PHC workforce internationally. Findings from this review highlight the need for action by managers, educators, employers and policy makers to enhance support for nurses in PHC.

### Implications for practice and research

There is urgent need to build capacity within the PHC nursing workforce internationally to meet service demands. This review has highlighted a number of issues around job satisfaction and career intention that impact on the retention of nurses in PHC. Exploring strategies to address the modifiable antecedents to nurse job dissatisfaction has the potential to improve retention. Maintaining happy and skilled nurses in the workforce has the potential to build workforce capacity and enhance patient outcomes.

This review has demonstrated that gaps remain in our knowledge around job satisfaction and career intention among PHC registered nurses. Further well-designed longitudinal research is required to explore the trajectory of careers in PHC. Additionally, mixed methods approaches are likely required to explore not only quantitative job satisfaction, but also to reveal how the aspects of satisfaction impact on PHC nurses.

## Abbreviation

PHC: Primary Health Care
